# *In Vitro* and *In Silico* Approaches for the Antileishmanial Activity Evaluations of Actinomycins Isolated from Novel *Streptomyces smyrnaeus* Strain UKAQ_23

**DOI:** 10.3390/antibiotics10080887

**Published:** 2021-07-21

**Authors:** Kamal A. Qureshi, Ibrahim Al Nasr, Waleed S. Koko, Tariq A. Khan, M. Qaiser Fatmi, Mahrukh Imtiaz, Riaz A. Khan, Hamdoon A. Mohammed, Mariusz Jaremko, Abdul-Hamid Emwas, Faizul Azam, Avinash D. Bholay, Gamal O. Elhassan, Dinesh K. Prajapati

**Affiliations:** 1 Faculty of Biosciences and Biotechnology, Invertis University, Bareilly 243123, UP, India; ka.qurishe@qu.edu.sa; 2 Department of Pharmaceutics, Unaizah College of Pharmacy, Qassim University, Unaizah 51911, Qassim, Saudi Arabia; go.osman@qu.edu.sa; 3 Department of Biology, College of Science and Arts, Qassim University, Unaizah 51911, Qassim, Saudi Arabia; insar@qu.edu.sa; 4 Department of Science Laboratories, College of Science and Arts, Qassim University, Ar Rass 51921, Qassim, Saudi Arabia; wa.mohamed@qu.edu.sa; 5 Department of Clinical Nutrition, College of Applied Health Sciences, Qassim University, Ar Rass 51921, Qassim, Saudi Arabia; tara.khan@qu.edu.sa; 6 Department of Biosciences, COMSATS University Islamabad, Islamabad 45600, Pakistan; qaiser.fatmi@comsats.edu.pk (M.Q.F.); mahrukhimtiaz92@yahoo.com (M.I.); 7 Department of Medicinal Chemistry and Pharmacognosy, College of Pharmacy, Qassim University, Buraydah 51452, Qassim, Saudi Arabia; ri.khan@qu.edu.sa (R.A.K.); ham.mohammed@qu.edu.sa (H.A.M.); 8 Biological and Environmental Sciences and Engineering Division (BESE), King Abdullah University of Science and Technology (KAUST), Thuwal 23955, Makkah, Saudi Arabia; mariusz.jaremko@kaust.edu.sa; 9 Core Labs, King Abdullah University of Science and Technology (KAUST), Thuwal 23955, Makkah, Saudi Arabia; abdelhamid.emwas@kaust.edu.sa; 10 Department of Pharmaceutical Chemistry and Pharmacognosy, Unaizah College of Pharmacy, Qassim University, Unaizah 51911, Qassim, Saudi Arabia; 11 Department of Microbiology, KTHM College, Savitribai Phule Pune University, Nashik 422002, MS, India; avinashbholay@kthmcollege.ac.in

**Keywords:** actinomycin X_2_, actinomycin D, *in silico* molecular modeling, Kala-azar, leishmaniasis, molecular dynamics simulation, *Streptomyces smyrnaeus*, strain UKAQ_23

## Abstract

Leishmaniasis, a Neglected Tropical Parasitic Disease (NTPD), is induced by several *Leishmania* species and is disseminated through sandfly (*Lutzomyia longipalpis*) bites. The parasite has developed resistance to currently prescribed antileishmanial drugs, and it has become pertinent to the search for new antileishmanial agents. The current study aimed to investigate the *in vitro* and *in silico* antileishmanial activity of two newly sourced actinomycins, X_2_ and D, produced by the novel *Streptomyces smyrnaeus* strain UKAQ_23. The antileishmanial activity conducted on promastigotes and amastigotes of *Leishmania major* showed actinomycin X_2_ having half-maximal effective concentrations (EC_50_), at 2.10 ± 0.10 μg/mL and 0.10 ± 0.0 μg/mL, and selectivity index (SI) values of 0.048 and 1, respectively, while the actinomycin D exhibited EC_50_ at 1.90 ± 0.10 μg/mL and 0.10 ± 0.0 μg/mL, and SI values of 0.052 and 1. The molecular docking studies demonstrated squalene synthase as the most favorable antileishmanial target protein for both the actinomycins X_2_ and D, while the xanthine phosphoribosyltransferase was the least favorable target protein. The molecular dynamics simulations confirmed that both the actinomycins remained stable in the binding pocket during the simulations. Furthermore, the MMPBSA (Molecular Mechanics Poisson-Boltzmann Surface Area) binding energy calculations established that the actinomycin X_2_ is a better binder than the actinomycin D. In conclusion, both actinomycins X_2_ and D from *Streptomyces smyrnaeus* strain UKAQ_23 are promising antileishmanial drug candidates and have strong potential to be used for treating the currently drug-resistant leishmaniasis.

## 1. Introduction

Leishmaniasis, a Neglected Tropical Parasitic Disease (NTPD), is induced by several species of *Leishmania* and transmitted through the bites of female phlebotomine sandflies [1,2,3,4]. In humans, the parasite lives and reproduces as an intracellular amastigote inside the macrophage phagolysosomes [3]. There have been three major kinds of leishmaniasis reported, i.e., cutaneous leishmaniasis, the most common form of leishmaniasis [5], induced by *L. donovani*, *L. aethiopica*, and *L. tropica*; mucocutaneous leishmaniasis, induced by *L. braziliensis*; and visceral leishmaniasis or Kala-azar, caused by *L. donovani*. The most severe form of leishmaniasis is visceral leishmaniasis [3]. Currently, no vaccine against human leishmaniasis is available [1], and chemotherapy is the only treatment option available for the affected population. Amphotericin B, paromomycin, and miltefosine are three commonly prescribed antileishmanial drugs, but their uses are restricted due to their toxicity or high costs. However, the existing drug repertoire is restricted, and increasing resistance to these drugs is a significant concern, especially in Saharan and sub-Saharan regions [1]. Therefore, discovering novel drug targets and evaluating novel drugs are essential for successful leishmaniasis control.

Over the last decade, there has been a paradigm shift in funding for anti-parasitic drug discovery. Certain organizations, including the Institute of One World Health (IOWH), the Drugs for Neglected Diseases Initiative (DNDi), and the Bill and Melinda Gates Foundation, have contributed funds towards the development of tropical disease drugs [1] by encouraging scientific advancements in academia and industry, such as publicly available gene sequencing, which has aided in the drug discovery and development processes. The whole-genome sequencing of several Leishmania species, i.e., *L. braziliensis*, *L. major*, and *L. infantum*, has contributed to significantly advancing drug discovery and development [6].

*Streptomyces*, a commercially treasured and medically significant actinomycetes genus, produces chemically diversified, biologically active substances, e.g., antibiotics, anticancer, antiviral, herbicidal, and insecticidal agents, which have heightened the interests in the genus and its microbial products [6,7,8]. The actinomycins are well-recognized antibiotics synthesized by various strains of the genus *Streptomyces*, which have exhibited anticancer and antimicrobial properties [7,8,9]. However, certain reports have suggested that actinomycins, including actinomycin D, also exhibit substantial levels of antileishmanial activity [10,11,12,13]. In this context, the current study aimed to isolate, purify, characterize, and evaluate the antileishmanial activity of two newly-sourced actinomycins, X_2_ and D, produced by the novel actinomycete strain *Streptomyces smyrnaeus* UKAQ_23. The study also explored, by the *in silico* methods, the molecular docking investigations of the isolated actinomycins against various antileishmanial target proteins, reported earlier through detailed analyses of the intermolecular interactions between the target and ligands, followed by molecular dynamics simulation studies and MMPBSA binding energy evaluations of the isolated actinomycins, X_2_ and D, obtained from the strain, UKAQ_23.

## 2. Results

### 2.1. Isolation, Identification, and Characterization of Strain UKAQ_23

**Figure 1:**
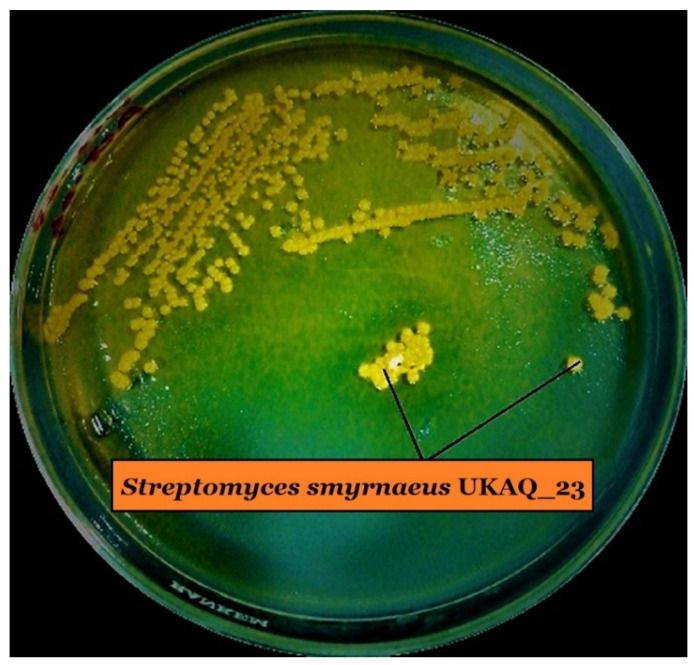
Growth of isolated strain UKAQ_23 on ISP-4 agar at 28 °C for 7 days.

The actinomycete strain, UKAQ_23, was isolated from a mangrove sediment sample collected from Jubail, Saudi Arabia, in 2019 (Figure 1), identified as *Streptomyces smyrnaeus* strain UKAQ_23, and comprehensively characterized. Our earlier published article has further details about this organism [14]. 

### 2.2. Isolation, Purification, and Characterization of Actinomycins X_2_ and D

**Figure 2:**
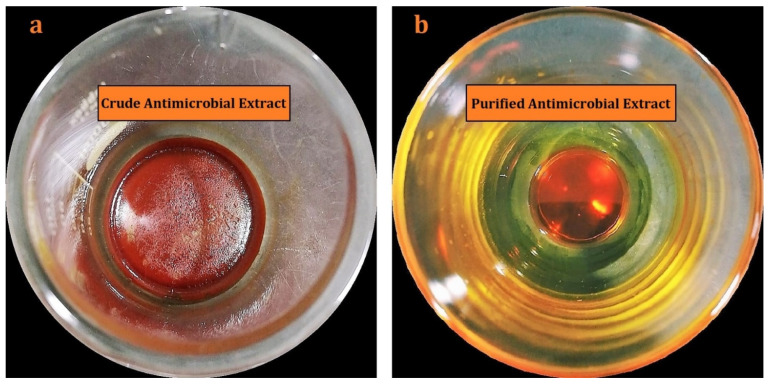
Antimicrobial extracts: (**a**) crude antimicrobial extract (**b**) purified antimicrobial extract.

The solid-liquid extraction technique yielded an amorphous, reddish-orange crude antimicrobial extract (Figure 2a). TLC showed two major components, K_1_, and K_2_ in the crude antimicrobial extract [14]. As a result, both the compounds were selected for further study. The pure components K_1_ and K_2_ were obtained by using various chromatographic techniques (Figure 2b). Additional information on the isolation, purification, and characterization of the actinomycins, X_2_ and D, are provided in the previously published article by us [14]. 

**Figure 3:**
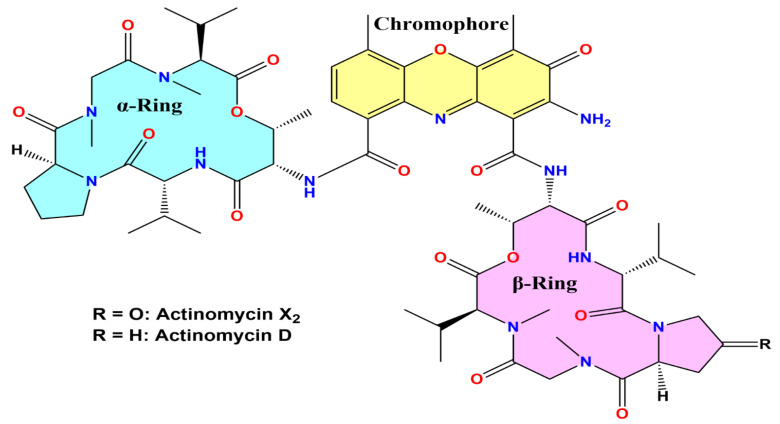
Structure of isolated actinomycin X_2_ and actinomycin D.

Structural confirmations, and *in vitro* and *in silico* antileishmanial activity evaluations of both the components, K_1_ and K_2_, were conducted. Thus, based on their physicochemical and spectroscopic analysis results, the isolated components K_1_ and K_2_ were identified as actinomycin X_2_ and actinomycin D, respectively (Figure 3). Additional information on the elucidation of the structures of the actinomycins, X_2_ and D, was provided in a previously published article by us [14]. 

### 2.3. Antileishmanial Activity Evaluations 

#### 2.3.1. Anti-Promastigotes Evaluations

**Figure 4:**
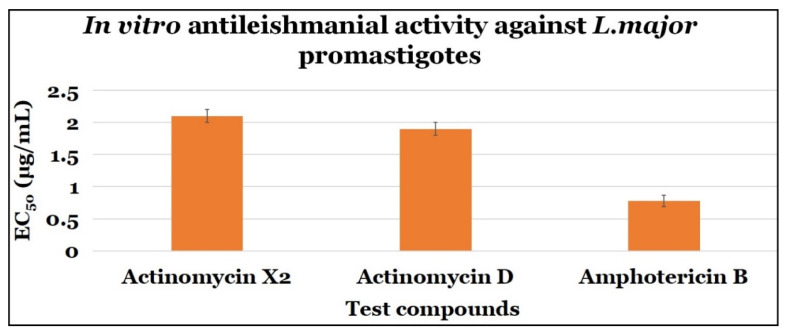
The anti-promastigotes activity of test compounds.

Both the actinomycins X_2_ and D demonstrated significant anti-parasitic activity against *L. major* promastigote stages, with EC_50_ values of 2.10 ± 0.10 μg/mL, and 1.90 ± 0.10 μg/mL with SI values of 0.048 and 0.053, respectively. Amphotericin B, the control drug, had an EC_50_ value of 0.78 ± 0.09 μg/mL and a SI value of 9.490 (Table 1 and Figure 4). 

#### 2.3.2. Anti-Amastigotes Evaluations

**Figure 5:**
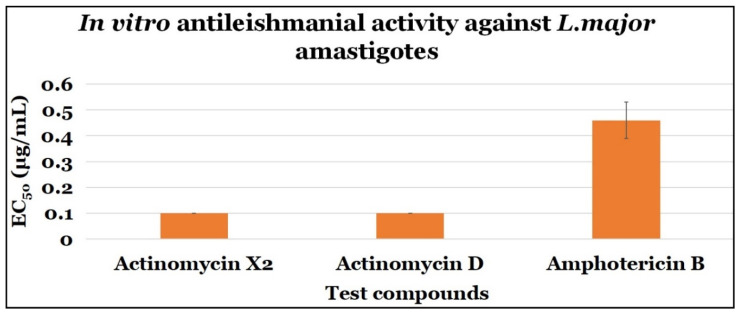
The anti-amastigotes activity of the tested compounds.

Both actinomycins, X_2_, and D, demonstrated significant anti-parasitic efficacy against *L. major* amastigote with EC_50_ values of 0.10 ± 0.0 μg/mL, and 0.10 ± 0.0 μg/mL, and SI values of 1 and 1, respectively. Amphotericin B, the control drug, had an EC_50_ value of 0.46 ± 0.07 μg/mL and a SI value of 16.09 (Table 2 and Figure 5).

The *in vitro* antileishmanial activity results showed that both the actinomycins, X_2_, and D, have significant antileishmanial activity, while actinomycin X_2_ exhibited significantly higher anti-promastigotes activity than the actinomycin D. 

#### 2.3.3. Statistical Analysis

There were no statistically significant differences in the mean antileishmanial activity values (*in vitro*) between the actinomycin X_2_ (M = 2.1, SD = 0.1) and actinomycin D (M = 1.9, SD = 0.1); t (4) = 2.25, *p* = 0.070. Additionally, the findings also indicated that the actinomycin X_2_ was somewhat more effective than the actinomycin D, since the *p* value (*p* = 0.070) was near to the statistical significance (*p* = 0.05).

### 2.4. In Silico Antileishmanial Activity Evaluations

#### 2.4.1. Molecular Docking Studies

To identify the target proteins for both the actinomycins, X_2_ and D, 15 previously reported druggable targets involved in several *L. major* metabolic pathways [1] were screened, except for the trypanothione reductase, which was from *L. infantum* [3]. 

The ligand-protein binding energy analysis was carried out, and the results are summarized in Table 3. Both the actinomycins, X_2_ and D, had the lowest predicted binding energy with squalene synthase, wherein actinomycin X_2_ predicted a slightly higher calculated affinity with this target protein, squalene synthase, which was consistent with the experimental results. The actinomycin X_2_ had slightly higher inhibitory activity than the actinomycin D. Several studies suggested that the squalene synthase was a potential target for *Leishmania donovani, Leishmania mexicana, Leishmania amazonensis*, and *Leishmania chagasi* [15,16,17,18].

**Figure 6:**
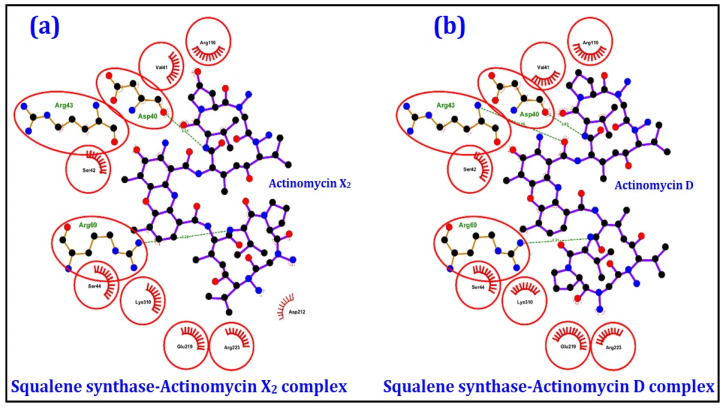
2D interactions analyses of the squalene synthase complexed with (**a**) actinomycin X_2_ and (**b**) actinomycin D, obtained from the molecular docking exercises. The AAs residues interacting with the ligand are shown in red circles.

**Figure 7:**
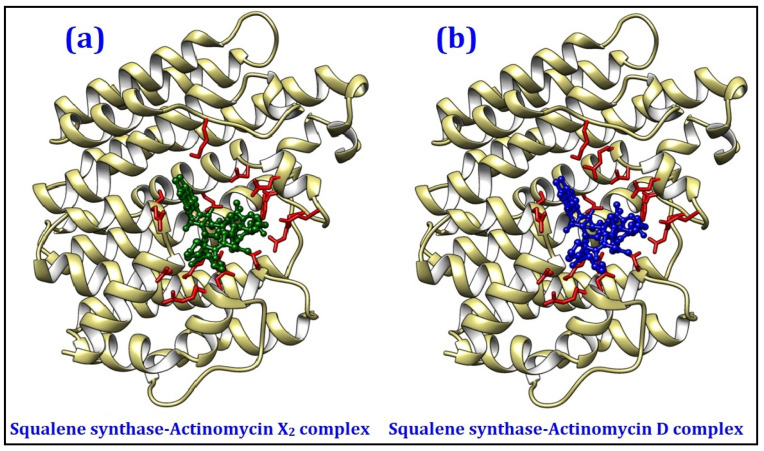
3D interaction analyses of squalene synthase complexed with (**a**) actinomycin X_2_, and (**b**) actinomycin D as generated by the molecular docking exercises.

The 2D and 3D ligand-protein interaction analyses for both the actinomycins, X_2_ and D, are given in Figure 6 and Figure 7, as obtained from Ligplot^+^ [19] and UCSF Chimera [20] tools, respectively. The results indicated that Val41, Ser42, Ser44, Arg110, Glu219, Arg223, and Lys310 were the common interacting residues in both the complexes, whose side chains predominantly form the hydrophobic interactions with the ligands. Similarly, Asp40, Arg43, and Arg69 residues were common in both complexes and were involved in hydrogen bonding with ligands. The remaining proteins exhibited poor to moderate binding affinities with both the actinomycins, X_2_ and D, and were not analyzed in detail.

#### 2.4.2. Molecular Dynamics (MD) Simulations

**Figure 8:**
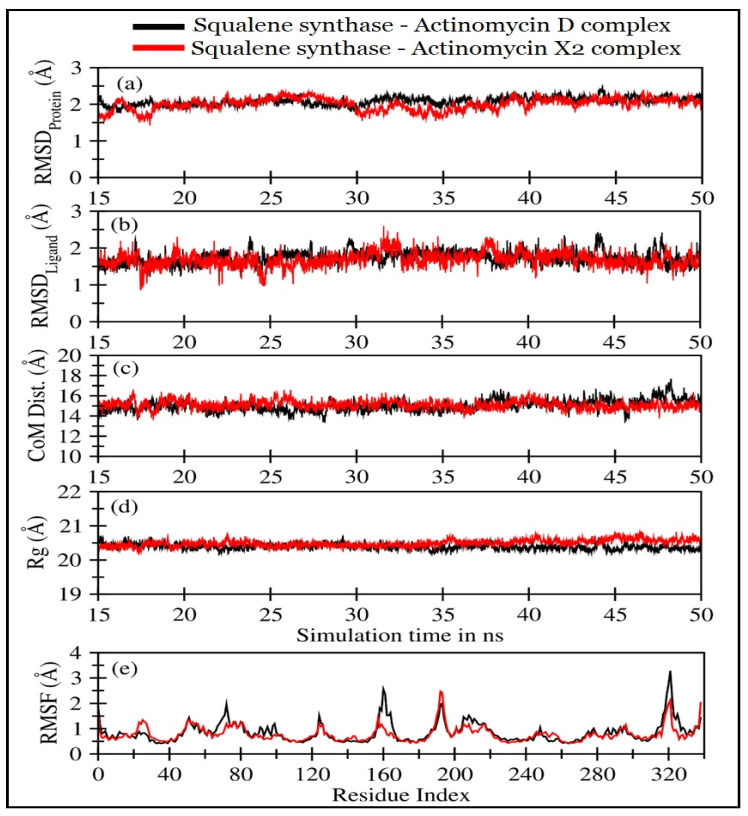
Various trajectory analyses for the squalene synthase complexed with actinomycin D (black lines) and actinomycin X_2_ (red lines): (**a**) RMSD_protein_, (**b**) RMSD_ligand_, (**c**) center-of-mass distance between protein and ligand, (**d**) protein’s radius of gyration, and (**e**) RMSF, as calculated from last 35 ns of MD trajectories. RMSD_protein_, Rg, and RMSF have been calculated using ‘C-alpha’ atoms using Gromacs or Bio3D modules in R programs.

The molecular dynamics simulations of actinomycins, X_2_ and D, in complexation with squalene synthase, were performed for 50 ns. The first 15 ns were discarded, and the remaining 35 ns trajectory was utilized to ensure that both the systems were completely converged. The trajectory analyses were performed in terms of root mean square deviation (RMSD) of protein and the molecular templates, actinomycins, X_2_ and D, root mean square fluctuations (RMSF), the radius of gyration (Rg), the center of mass distance (COM) between the protein and the actinomycins, X_2_ and D (Figure 8), and the number of H-bonds, and MMPBSA [21] ligand-protein binding energy (Figure 8). 

Figure 8a displayed RMSD for squalene synthase complexed with the actinomycin X_2_ (1.92 ± 0.25 Å), and actinomycin D (1.96 ± 0.27 Å) (Table 4). The actinomycin X_2_ showed slightly less deviation in comparison with the actinomycin D. However, both the systems not only displayed RMSD values less than 2 Å but also exhibited highly stable RMSD in the last 35 ns of simulations, indicating that these systems remained stable throughout the simulations. No significant deviations were observed for both the systems involving actinomycins, X_2_ and D. The RMSD values (Figure 8b) and their mean values for both the ligands are listed in Table 3. It was also observed that the actinomycin X_2_ showed slightly less RMSD_Ligand_ value (1.52 ± 0.30 Å) with a smaller SD in the binding pocket of the squalene synthase than that observed for the actinomycin D (1.58 ± 0.31 Å). Nonetheless, both the ligands remained stable throughout the simulation period with some slight fluctuations within the binding pockets, which did not alter the stability of the ligands systems.

As shown in Figure 8c and Table 4, the center-of-mass distance represented the distance between the center-of-mass of the protein and the center-of-mass of the ligand during MD simulations. It was observed that both the distances remained highly stable throughout the simulation period, representing that both the ligands and the proteins remained in the bound state. However, the actinomycin X_2_ displayed slightly closer binding with the squalene synthase than the actinomycin D. Figure 8d showed the radii of gyration plots of the squalene synthase in the presence of actinomycins D and X_2_; the mean values are provided in Table 4. The radius of gyration indicated the compactness of the protein during simulation time, and no significant changes were observed in protein compactness for both the ligands systems involving actinomycin D and actinomycin X_2_ with values at 20.40Å and 20.48Å, respectively, thereby indicating that both the ligands did not induce any significant changes in the protein folds. The root means square fluctuations (RMSF) for Cα atoms of the squalene synthase, in both the systems, as shown in Figure 8e, indicated that the AAs residues 155–170, and AAs residues 315–330 (representing short helices near the binding pocket) were more flexible in the case of actinomycin D as compared with the actinomycin X_2_. These observations supported that the actinomycin D ligand complex had a slightly higher RMSD value. Overall, the ligands’ fluctuating patterns were similar, wherein the C-terminal exhibited slightly higher fluctuations than the N-terminal of the binding protein.

**Figure 9:**
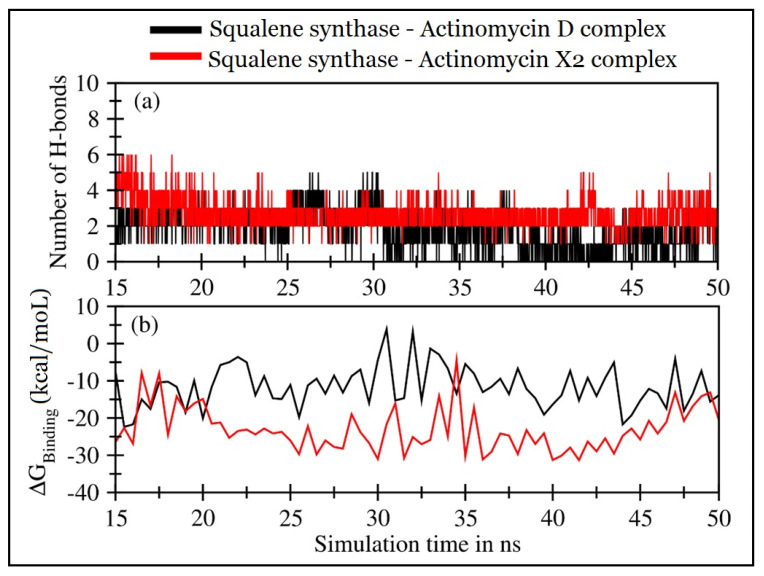
Trajectory analyses for the squalene synthase complexed with actinomycins D (black lines) and X_2_ (red lines): (**a**) number of hydrogen-bonds formed between the protein and the actinomycins D (mean: 2.02 ± 1.10) and X_2_ (mean: 2.99 ± 1.04), and (**b**) molecular mechanistic Poisson–Boltzmann surface area protein–ligand binding energy calculated after every 0.5 ns.

Figure 9a displayed the hydrogen bonds formed between the squalene synthase and the corresponding ligands. Both the actinomycins, X_2_ and D, systems showed consistent H-bond formations. However, the squalene synthase-actinomycin X_2_ complex showed consistent H-bonds involving AAs residues Val41, Ser42, and Arg69, as depicted in Figure S1. In contrast, the AAs Arg43 and Arg313 showed non-consistent H-bonds with the ligand as compared with the squalene synthase-actinomycin D complex, wherein the mean number drops to ~2, and only the AA Ser42 residue was consistent, while Arg69, and Arg313, either form H-bond in the beginning or later in the simulation period. Figure S1 also supported that the RMSD_Ligand_ and the RMSF were slightly lowered in the case of the actinomycin X_2_ complex. The MMPBSA protein-ligand binding energy was calculated after every 0.5 ns of simulations for both systems. 

Figure 9b displayed the binding energies over the simulation’s period, and Table 5 lists the mean values and individual contributions from van der Waal’s electrostatic and polar and non-polar solvation energies. 

Additionally, actinomycin X_2_ predicted a stronger calculated binding affinity with squalene synthase with a mean binding energy value of −23.07 ± 5.96 kcal/mol than the actinomycin D complex mean energy values at −11.57 ± 5.53 kcal/mol. The significant energy contributions that favored the binding of actinomycin X_2_ with squalene synthase came from Coulombic interactions and were substantiated by higher H-bond formations between the protein and the actinomycin ligand. Briefly, both the ligands exhibited fair to moderate binding interactions with the squalene synthase. However, actinomycin X_2_ demonstrated the potential to be a far more active drug than actinomycin D.

## 3. Discussions

Leishmaniasis, being among the most prevalent diseases in several countries with continuously increasing morbidity and mortality, has posed challenges to health programs and the well-being of populations. The fact that a few drugs are available has made the situation grimmer. The drugs’ toxicity, resistance in treatments, and the availability and cost of the drugs are other concerns. The drug discovery through finding new molecular templates from natural sources has been one of the options. The target identification, molecular docking, binding feasibility, and activity validation through *in silico*, *in vitro*, and *in vivo* approaches have been performed. To establish a successful pipeline for new drug discovery and development, it is necessary to discover new antileishmanial active compounds from the novel sources, novel chemistry, and adaptable appropriate mechanisms of action. The drugs repurposing, structure-, and fragments-based drug designs, chemistry and bioinformatics tools, biological chemistry approaches, structural genomics, and molecular dynamics are some of the newer technological advancements for new drug discovery against several diseases.

Our approach in discovering and developing effective and affordable antileishmanial drugs utilized *in silico* and *in vitro* evaluations of the naturally-sourced molecular templates obtained from the *Streptomyces smyrnaeus* strain UKAQ_23. Supposedly, the low translational outcomes from the *in vitro* assays were validated by *in silico* conditions. The thorough and in detail approach to the ligands binding, energy estimations, and geometric preferences outcomes together with the stability of the bound ligand and the target, involvement of amino acids residues facilitating the bindings, and overall energy requirements provided the needed comparison between the predicted efficacy of actinomycins X_2_ and D bindings led activity projection. The relationship with the *in vitro* investigations conducted on the isolated products, actinomycin X_2_, and actinomycin D, indicates the active components between the two targeted compounds isolated during the current study. Hence, the chances of attrition were minimized, and the prospects of the isolated compounds were more relevant to fit the new drug discovery. The molecular dockings against various ligand proteins from two different *Leishmania* species, *L. major* and *L. infantum*, were utilized. A total of 15 enzymes from trypanothione, sterol biogenetic, glycolytic, hypusine biosynthetic, and purine salvage pathways were utilized. The *in silico* evaluations on squalene synthase from the sterol biogenetic pathway produced the potent interaction towards bindings of the ligands. Molecular dynamics trajectory analyses based on RMSD (root mean square deviation) of the target and the molecular templates, actinomycins, X_2_ and D, as well as the RMSF (root mean square fluctuations), Rg (radius of gyration), COM (center of mass distance) between the protein and the actinomycins, X_2_ and D, the number of H-bonds, and MMPBSA ligand-protein binding energy were estimated. 

Our findings on actinomycins, X_2_, and D, produced by *Streptomyces smyrnaeus*, strain UKAQ_23, are consistent with the previous findings that several *Streptomyces* strains, including *Streptomyces* sp. MS449, *Streptomyces* sp. IMB094, *Streptomyces nasri* YG62, *Streptomyces padanus* JAU4234, *Streptomyces elizabethii*. II, *Streptomyces flavogriseus* NJ-4, *Streptomyces* MITKK-103, *Streptomyces griseoruber*, *Streptomyces* strain M7, *Streptomyces* sp. HUST012, *Streptomyces heliomycini*, *Streptomyces hydrogenans* IB310 produce actinomycin X_2_ and actinomycin D, which have provided anti-parasitic and some with anti-leishmanial compounds [14,22,23,24,25,26,27,28,29,30,31,32,33]. Several previously published reports specified that actinomycins D, Z3, Z5 have significant antileishmanial activity [10,11,34]. Jamal reported the antileishmanial activity of actinomycins D, Z3, and Z5 against promastigotes and amastigotes of *L. tropica* and their cytotoxicity towards human peripheral blood lymphocytes. It was also reported that the IC_50_ values for the antileishmanial activity of actinomycins D, Z3, and Z5 were at 8.739 μM, 2.135 μM; 5.500 μM, 1.760 μM; 9.529 μM, 1.691 μM concentrations, respectively, against promastigotes and amastigotes of *L. tropica,* while the cytotoxicity (IC_50_ values) of the actinomycins D, Z3, and Z5 were at 195.8 μM, 210.1 μM, and 234.9 μM concentrations, respectively, against human peripheral blood lymphocytes [10]. These findings are consistent with our results, which demonstrated substantial antileishmanial activity of actinomycin X_2_ and D. Another report by Annang et al. found antileishmanial activity (IC_50_) of actinomycin D against *L. donovani* to be at 147.9 nM concentration, which is also in agreement with the results obtained in the present study [11], while Kaplum et al. reported the antileishmanial activity (IC_50_) of actinomycin D against promastigotes of *L. amazonensis* to be at 50 μM concentration, again consistent with our findings, indicating antileishmanial activity of actinomycin D [34]. 

## 4. Materials and Methods

### 4.1. Isolation, Identification, and Characterization of Strain UKAQ_23

The mangrove sediment sample was collected from Jubail, Saudi Arabia (latitude: 27°00′40″ N; longitude: 49°39′29″ E; altitude: 22 ft; annual rainfall: 97 mm; average temperature: 26.6 °C) by following the standard techniques [35,36]. An actinomycete strain, UKAQ_23, was isolated from the collected mangrove sample by following the standard methods [35,36]. Our previously published article described further details about this organism [14].

### 4.2. Production, Purification, and Characterization of Actinomycins 

The production of actinomycins was conducted on a modified ISP-4 agar medium at pH 6.5 ± 0.1, temperature 35 ± 1 °C, inoculum 5% (*v*/*w*), and time duration of 7 days by following the solid-state fermentation [14,37,38]. 

The antimicrobial extract containing the actinomycins was extracted from the fermented agar using the solid-liquid extraction technique and subsequently purified using various chromatographic techniques [14].

For the physicochemical characterization and structural determination of actinomycins X_2_ and D, we used the following techniques: color, appearance, solubility, melting point (°C), UV-Visible (λ_max_, nm) absorbance, FT-IR (υ_max_, cm^−1^) absorbance, monoisotopic masses, (+)-HR-ESI-MS, LC-MS, LC-MS-MS, 1D, and 2D NMR spectroscopy. An earlier published article by us described the structure elucidation of the actinomycins X_2_ and D [14]. 

### 4.3. In Vitro Antileishmanial Evaluations 

#### 4.3.1. Isolation of *L. major* and Culture Conditions 

The parasite was isolated as promastigotes of *L. major* from a male patient and cultivated weekly at 26 °C in Schneider’s Drosophila medium (SDM) supplemented with 10% (*v*/*v*) heat-inactivated fetal bovine serum (FBS). Promastigotes were cryopreserved at a concentration of 3 × 10^6^ parasites/mL in liquid nitrogen. To maintain virulence, the parasite in the promastigote form (1 × 10^6^) was injected into the hindfoot of female BALB/c mice, and then amastigotes were isolated from mice after 8 weeks. Amastigotes were converted into promastigotes at a temperature of 26 °C using SDM containing 10% (*v*/*v*) FBS and antibiotics. Promastigotes (<5 *in vitro* cycles) were then used for infection and anti-parasitic studies. Mice were kept in a pathogen-free environment [2,3,4].

#### 4.3.2. Anti-Promastigotes Evaluations

The promastigotes with logarithmic-phase were cultured in Roswell Park Memorial Institute (RPMI)-1640 medium (phenol red-free and supplemented with 10% (*v*/*v*) FBS) and then transferred into 96-welled microtiter plates with a density of 10^6^ cells/mL (200 µL/well). The parasites were counted using a hemocytometer. The anti-promastigotes efficacy of test actinomycins and the control drug was evaluated at different concentrations, including 25, 8.3, 2.7, 0.9, 0.3, and 0.1 µg/mL. Amphotericin B and dimethyl sulphoxide (DMSO) were used as positive and negative controls, respectively. After 72 h of incubation, the parasite inhibition was determined by counting viable promastigotes using a tetrazolium dye (MTT)-based colorimetric technique. The samples were analyzed at 540 nm using an ELISA reader. Each test was performed in triplicate, and the findings were recorded in mean ± SD [2,3,4]. 

#### 4.3.3. Anti-Amastigotes Evaluations

The anti-amastigotes efficacy of the test compounds was assessed by following a previously published method [2,3,4]. Female BALB/c mice aged 6–8 weeks were used to harvest the peritoneal macrophages. The cells (5 × 10^4^) were then cultured in RPMI-1640 medium containing 10% (*v*/*v*) FBS in 96-welled microtiter plates for 4 h at 37 °C with 4% CO_2_ to promote the cell attachment. After discarding the media, the attached macrophages were washed with PBS and then incubated with 200 µL of RPMI-1640 medium (phenol red-free) supplemented with 10% (*v*/*v*) FBS containing promastigotes of *L. major* in a ratio of 10 promastigotes: 1 macrophage. After that, the plates were incubated at 37 °C with 5% CO_2_ for 24 h to allow amastigotes to infect and differentiate. The test compounds, including a positive control, were then added to the infected macrophages and 200 µL of RPMI-1640 medium (phenol red-free) containing 10% (*v*/*v*) FBS. After 3 washes with PBS to remove free promastigotes, a serial dilution was carried out to obtain final concentrations of 25, 8.3, 2.7, 0.9, 0.3, and 0.1 µg/mL for each of the test compounds and the positive control. The microtiter plates were then incubated for 72 h at 37 °C with 5% CO_2_. The cultures with DMSO served as the negative control, while cultures with amphotericin B at the same concentrations as test actinomycins served as the positive control. After washing, methanol fixation, and Giemsa staining, the percentage of infected macrophages was assessed microscopically. Each test was conducted in triplicate, and the findings were recorded in mean ± SD [2,3,4]. 

#### 4.3.4. In Vitro Cytotoxicity Evaluations 

The MTT assay was performed to assess the cytotoxicity of actinomycins, X_2_, and D. The mice macrophages were cultivated for 24 h in RPMI-1640 medium supplemented with 10% (*v*/*v*) FBS in microtiter plates (5 × 10^3^ cells/well/200 μL) at 37 °C with 5 % CO_2_. After washing the cells with PBS, cells were treated with actinomycins, X_2_, and D for 72 h at differing concentrations, i.e., 25, 8.3, 2.7, 0.9, 0.3, and 0.1 µg/mL in RPMI-1640 medium supplemented with 10% (*v*/*v*) FBS. The cells with 10 % (*v*/*v*) FBS served as the negative control. After removing the supernatant, a mixture of 50 μL RPMI-1640 medium and 14 μL of MTT (0.5% *w*/*v*) was dispensed and incubated for 4 h. After removing the supernatant, 200 μL DMSO was used to solubilize the insoluble formazan produced by living cells from MTT. The colorimetric analysis was performed using the Bio-Rad X-Mark microplate reader at 540 nm. The cytotoxic effects were quantified using CC_50_ values (the concentration at which 50% of viable cells were killed). Each test was performed in triplicate, and the findings were recorded in mean ± SD [2,3,4]. 

#### 4.3.5. Statistical Analysis

Microsoft Excel was used to calculate the linear regression equation for EC_50_ and CC_50_ values. The SI values were calculated by dividing the CC_50_ by the EC_50_ for each parasite. The significance of the variations in means between actinomycin X_2_ and actinomycin D were calculated using an independent-samples (unpaired) *t*-test, with a significance level of *p* = 0.05. SPSS software, version 20.0 (IBM, USA), was used to conduct the statistical analyses [1,2,3,4].

### 4.4. In Silico Antileishmanial Evaluations 

#### 4.4.1. Molecular Docking Evaluations

The UniProt was used to obtain the amino acid sequences of all of the proteins. The 3D structures were either retrieved from PDB and/or model from the Swiss model or I-tasser, depending upon the availability of the template proteins and their percent identity. The FASTA sequences of all the proteins are given in Table S1. After validation of all the structures, the .pdbqt files were generated using AutoDock tools [37] employing Kollman charges. The grid box in x, y, and z dimensional domains were defined to cover the whole protein to perform blind docking. The grid spacing was set to 1 Å, as recommended for the AutoDock vina program [38]. The 3D structure of actinomycin D was retrieved from PDB (PDB entry code: 1A7Y) [39], and it was further modified to generate the structure of actinomycin X_2_. Before the preparation of .pdbqt files using Gasteiger charges, both the ligands were energy minimized. Finally, the molecular dockings were run utilizing the AutoDock vina program [38] using an exhaustiveness value of 80. A total of 20 docked conformations were generated for each system for analysis.

#### 4.4.2. Molecular Dynamics Simulations

The MD simulations of squalene synthase complexes were performed for 50 ns using the GROningen MAchine for Chemical Simulations (GROMACS) simulation software (GROMACS 2020.4; Department of Biophysical Chemistry, University of Groningen, Groningen, The Netherlands and the Royal Institute of Technology and Uppsala University, Sweden) [40], with the Chemistry at HARvard Macromolecular Mechanics 36m (CHARMM36m) forcefield for proteins, and the CHARMM General Force Field (CGenFF) for actinomycins, X_2,_ and D. The trajectory and energy data were recorded at every 10 ps. The TIP3P water molecules were used to solvate both the systems in a truncated octahedral box. The protein complexes were set in the simulation boxes within 10 Å from the box edge to accurately meet the minimum image convention. Additionally, 12 K^+^ ions were added to each of the squalene synthase complexes to neutralize the whole system. The systems for actinomycins, X_2_ and D, contained 46,122 and 46,153 atoms, respectively. The Chemistry at HARvard Macromolecular Mechanics-Graphical User Interface (CHARMM-GUI) webserver was used to generate all input files [41,42].

The system was minimized for 5000 steps using the Steepest Descent technique, and convergence was achieved under the force limit of 1000 (kJ/mol/nm) to exclude any steric disturbances. Finally, the system was equilibrated at NVT (Canonical ensemble: where moles, N; volume, V; and temperature, T were conserved) and NPT (Isothermal-Isobaric ensemble: where moles, N; pressure, P; and temperature, T were conserved) ensembles for 100 ps (50,000 steps) and 1000 ps (1,000,000 steps), respectively, using time steps 0.2 and 0.1 fs, at 300 K to ensure a fully converged system for the production run.

The simulation runs were conducted at a constant temperature of 300 K and a pressure of 1 atm, or 1 bar (using an NPT ensemble), respectively, utilizing weak coupling velocity rescaling (modified Berendsen thermostat) and Parrinello–Rahman algorithms. The relaxation periods were set at τ T = 0.1 ps and τ P = 2.0 ps. Using the LINear Constraint Solver (LINCS) algorithm, all bond lengths involving hydrogen atoms were maintained stiffly at optimal bond lengths, with a time step of 2 fs. The non-bonded interactions were calculated using the Verlet technique. In all x, y, and z directions, Periodic Boundary Conditions (PBC) were applied. Each time, interactions within a short-range cutoff of 12 Å were determined. The electrostatic interactions and forces in a homogeneous medium beyond the long-range cutoff were calculated using the Particle Mesh Ewald (PME) method. For both the complexes, the production ran for 50 ns. The first 15 ns of the trajectories were excluded for comprehensive analysis, and the remaining 35 ns were utilized.

## 5. Conclusions

The drug-resistant leishmaniasis is becoming more frequent by compromising the therapeutic efficacy of the currently available antileishmanial drugs, thereby emphasizing the urgent need for finding new antileishmanial drugs with enhanced activity to treat drug-resistant leishmaniasis. In this perspective, several pieces of evidence indicated that terrestrial actinomycetes are among the most promising candidates for producing novel bioactive molecules active against a range of pathogenic microorganisms, including the *Leishmania* parasite. As a result, the isolation of biologically active molecules from terrestrial actinomycetes was undertaken. The current study pursued the antileishmanial activity of actinomycins X_2_ and D, isolated from *Streptomyces smyrnaeus* strain UKAQ_23, against promastigotes and amastigotes of *Leishmania major*. The actinomycin X_2_ showed higher antileishmanial efficacy than the actinomycin D. However, both the products demonstrated substantial antileishmanial activity against the promastigotes and amastigotes of *L. major* determined in the *in vitro* experimentations and validated through the *in silico* predictions. In conclusion, both the actinomycins X_2_ and D can be utilized to treat drug-resistant leishmaniasis, and *Streptomyces smyrnaeus* strain UKAQ_23 has the potential to be a commercially viable source for the production of actinomycins X_2_ and D, probably implying a paradigm shift in the pharmaceutical industry.

## 6. Patents

On 22 January 2021, a patent application has been submitted to the Intellectual Property Office of India. The reference number for the patent filing is 202111003185. The final decision is awaited.

## Figures and Tables

**Table 1:** Anti-promastigotes activity of tested compounds.

Compounds	Anti-Promastigotes Evaluation		
	EC_50_ (μg/mL)	CC_50_ (μg/mL)	SI
Actinomycin X_2_	2.10 ± 0.10	0.10 ± 0.0	0.048
Actinomycin D	1.90 ± 0.10	0.10 ± 0.0	0.053
Amphotericin B	0.78 ± 0.09	7.40 ± 2.64	9.490

Note: The results are demonstrated in mean ± SD. Each test was performed in triplicate.

**Table 2:** Anti-amastigotes activity of tested compounds.

Compounds	Anti-Amastigotes Evaluation		
	EC_50_ (μg/mL)	CC_50_ (μg/mL)	SI
Actinomycin X_2_	0.10 ± 0.0	0.10 ± 0.0	1.0
Actinomycin D	0.10 ± 0.0	0.10 ± 0.0	1.0
Amphotericin B	0.46 ± 0.07	7.4 ± 2.64	16.09

Note: The results are demonstrated in mean ± SD. Each test was performed in triplicate.

**Table 3:** Protein-ligand predicted binding energy obtained from molecular dockings.

Enzymes	Pathway	Binding Energy (kcal/mol)	
		Actinomycin X_2_	Actinomycin D
Trypanothione reductase	Trypanothione pathway	−8.8	−8.8
Trypanothione synthetase-amidase	Trypanothione pathway	−8.5	−8.1
Tryparedoxin peroxidase	Trypanothione pathway	−8.0	−8.1
Squalene synthase	Sterol biogenetic pathway	−10.0	−9.9
Squalene monooxygenase	Sterol biogenetic pathway	−7.5	−7.3
Farnesyl pyrophosphate synthase	Sterol biogenetic pathway	−8.3	−8.0
Glyceraldehyde-3-phosphate dehydrogenase	Glycolytic pathway	−7.7	−7.5
Triosephosphate isomerase	Glycolytic pathway	−6.8	−6.7
Phosphoglycerate kinase	Glycolytic pathway	−8.0	−8.1
Pyruvate kinase	Glycolytic pathway	−8.0	−7.7
Phosphoglycerate mutase (2,3-diphosphoglycerate-independent)	Glycolytic pathway	−7.9	−7.8
Fructose-bisphosphate aldolase	Glycolytic pathway	−8.7	−8.7
Adenine phosphoribosyltransferase	Purine salvage pathway	−7.6	−7.3
Xanthine phosphoribosyltransferase	Purine salvage pathway	−8.8	−5.9
Deoxyhypusine hydroxylase	Hypusine biosynthetic pathway	−8.5	−8.4

**Table 4:** Mean standard deviation values for RMSD_Protein_, RMSD_Ligand_, center of the mass distance between protein–ligand (CoM_Protein-Ligand_) and Rg for squalene synthase complexed with actinomycin X_2_ and actinomycin D ligands.

Squalene Synthase Complexed with:	RMSD_Protein_ (Å)	RMSD_Ligand_ (Å)	CoM_Protein-Ligand_ (Å)	Rg (Å)
Actinomycin X_2_	1.92 ± 0.25	1.52 ± 0.30	14.80 ± 0.69	20.48 ± 0.1
Actinomycin D	1.96 ± 0.27	1.58 ± 0.31	15.05 ± 0.57	20.40 ± 0.09

**Table 5:** MMPBSA binding energy in kcal/mol for the protein-ligand complex.

Squalene Synthase Complexed with:	∆E^VDW^ (Van der Waal’s Energy)	∆E^elec^ (Coulombic Energy)	∆G^PB^ (Poisson-Boltzmann Polar Solvation Energy)	∆E^SASA^ (Non-Polar Solvation Energy)	∆G^MMPBSA^ (Protein-Ligand Binding Energy)
Actinomycin X_2_	−45.14 ± 5.33	−35.37 ± 10.16	62.93 ± 10.62	−5.49 ± 0.38	−23.07 ± 5.96
Actinomycin D	−41.78 ± 5.217	−12.31 ± 11.48	47.95 ± 11.05	−5.42 ± 0.38	−11.57 ± 5.53

## Data Availability

The data presented in this study are partially available at https://doi.org/10.1038/s41598-021-93285-7, and the complete data can be obtained upon request from the corresponding authors.

## Supplementary Materials

The following are available online at https://www.mdpi.com/article/10.3390/antibiotics10080887/s1, Figure S1: H-bond distances calculated between heavy atoms of protein (i.e., O or N) and ligand (i.e., O or N) for (a) actinomycin D and (b) actinomycin X_2_ complexes. The hydrogen bonds were calculated using VMD, where the cutoff for Donor-Acceptor distance and angle was set to 3 Å and 20 degrees, respectively, Table S1: The selected proteins for molecular docking studies with actinomycin D and actinomycin X_2_ ligands targeting multiple pathways in *Leishmania major*. The table also lists the name of enzymes, their FASTA sequence, amino acid length, their PDB code (if existed) or models (generated from homology modeling approach or threading approach), and the corresponding pathways where the protein is involved.
