# Supplementary material for: In Vitro and In Silico Approaches for the Antileishmanial Activity Evaluations of Actinomycins Isolated from Novel Streptomyces smyrnaeus Strain UKAQ_23
Source: Antibiotics (Basel). 2021 Jul 21;10(8):887. doi: 10.3390/antibiotics10080887 (PMC8388687; doi:10.3390/antibiotics10080887)
Supplement: Supplementary file 1 [file antibiotics-10-00887-s001.zip › antibiotics-1284691-supplementary.pdf]

*antibiotics*

MDPI

*Supplementary Information*

*Article*

# *In Vitro* and *In Silico* Approaches for the Antileishmanial Activity Evaluations of Actinomycins Isolated from Novel *Streptomyces smyrnaeus* Strain UKAQ_23

**Kamal A. Qureshi [1,2], Ibrahim Al Nasr [3,4], Waleed S Koko [4], Tariq A Khan [5], M. Qaiser Fatmi [6], Mahrukh Imtiaz [6], Riaz A Khan [7], Hamdoon A Mohammed [7], Mariusz Jaremko [8], Abdul-Hamid Emwas [9], Faizul Azam [10,*], Avinash D. Bholay [11], Gamal O. Elhassan [2] and Dinesh K. Prajapati [1,*]**

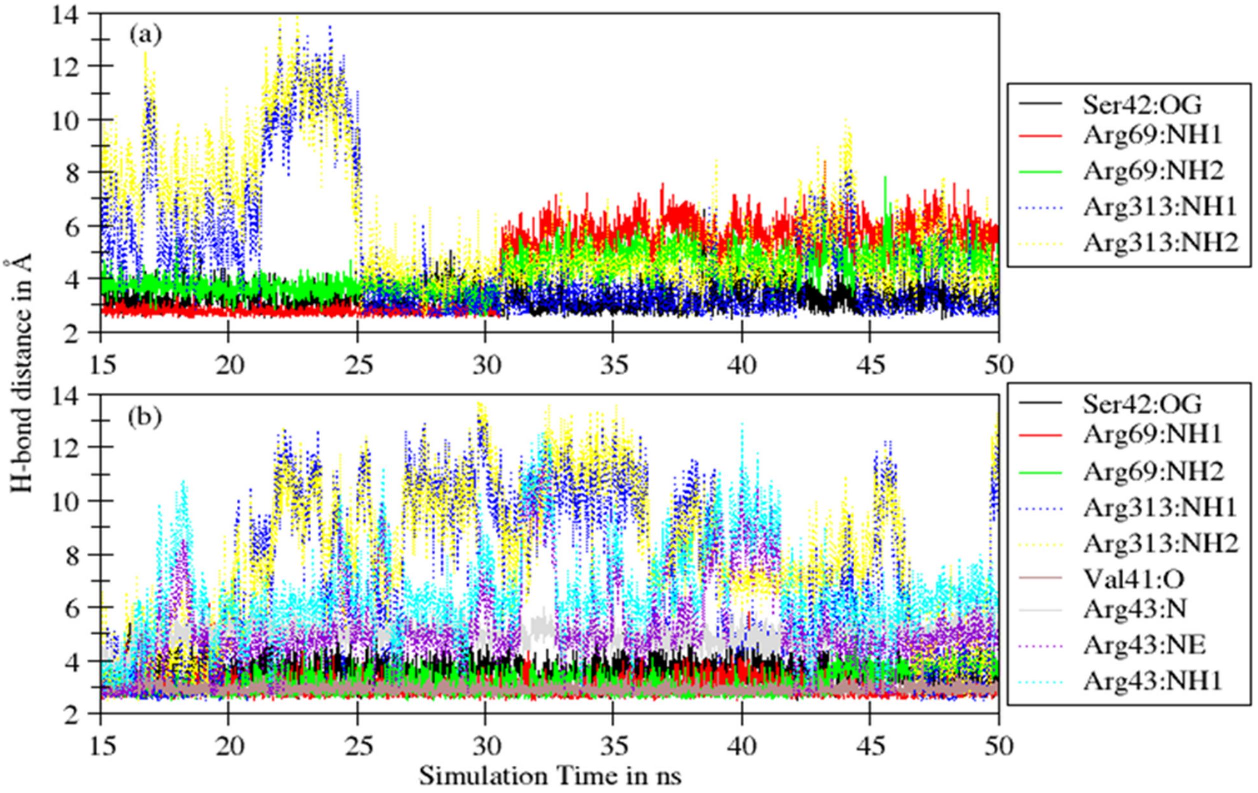


**Figure S1.** H-bond distances calculated between heavy atoms of protein (*i.e.*, O or N) and ligand (*i.e.*, O or N) for (a) actinomycin D and (b) actinomycin $X_2$ complexes. The hydrogen bonds were calculated using VMD, where the cutoff for Donor-Acceptor distance and angle was set to 3 Å and 20 degrees, respectively.

**Table S1.** The selected proteins for molecular docking studies with actinomycin D and actinomycin $X_2$ ligands targeting multiple pathways in *Leishmania major. The table also lists the name of enzymes, their FASTA sequence, amino acid length, their PDB code (if existed) or models (generated from homology modeling approach or threading approach), and the corresponding pathways where the protein is involved.*

| Enzyme | Organism | FASTA | Amino acid | PDB/Model | Pathway |
|---|---|---|---|---|---|
| Trypanothione reductase | *Leishmania infantum* | MGSSHHHHHHSSGLVPRGSHMSRAYDLV<br>VLGAGSGGLEAGWNAAVTHKKKVAVVD<br>VQATHGPPLFAALGGTCVNVGCVPKKLM<br>VTGAQYMDLIRESGGFGWEMDRESLCPN<br>WKTLIAAKNKVVNSINESYKSMFADTEGL<br>SFHMGFGALQDAHTVVVRKSEDPHSDVL<br>ETLDTEYILIATGSWPTRLGVPGDEFCITSN<br>EAFYLEDAPKRMLCVGGGYIAVEFAGIFN<br>GYKPCGGYVDLCYRGDLILRGFDTEVRKS<br>LTKQLGANGIRVRTNLNPTKITKNEDGSN<br>HVHFNDGTEEDYDQVMLAIGRVPRSQAL<br>QLDKAGVRTGKNGAVQVDAYSKTSVDNI<br>YAIGDVTNRVMLTPVAINEGAAFVETVFG<br>GKPRATDHTKVACAVFSIPPIGTCGMTEEE<br>AAKNYETVAVYASSFTPLMHNISGSKHKE<br>FMIRIITNESNGEVLGVHMLGDSAPEIIQSV<br>GICMKMGAKISDFHSTIGVHPTSAEELCS<br>MRTPAYFYESGKRVEKLSSNL | 511 | 2JK6 | Trypanothione pathway |
| Trypanothione synthetase-amidase | *Leishmania major* | MSSLQRASVSFNKPGHIPFGAVQGYAPGG<br>VPAYSNKHDHYFSGERNIEDNIFFGFKYQ<br>CVEFARRWLLVRKGLLLPDVNWACHIFQ<br>LKEVRDAATTESFAVLQVRNGTTTKPEAD<br>ALLVYPSTDANPVGHVGTITEVGDDYVCV<br>ADQNYRFHKWESSCAYKLKLDHRDGIWT<br>IIDDIDADEIEIPLGWLTFPGRANRPEGAPP<br>VALHPSLHFKEPPKPYLLRRNFLPTESKAN<br>WLDMNNPAERLFVEEFGMDVSRTRLEEK<br>VVSYYESNHEFHLRCVAYGTQLHAIFMEA<br>TAQVIESDEKLRLFAIPEEFWPRIRHSWKY<br>QQTYISGRFDFAFNNETGEVKCFEYNADS<br>ASTLLECGLIQQKWAESVGLDKQDTRGSG<br>FAVERNLKMAWANSGATGRVHFCVDEE<br>REEQYTALYCMQAAEAVGLEGKLCILFDE<br>FRFDDNGHVVDSDGVRVRNVWKTWMW<br>ESAITDYYAAREERGENWKPSPKDKVRLC<br>DLLLGDDWEILYFEPMWKVIPSNKAILPMI<br>YHNHPEHPAILKAEYELTDELRKHGYAK<br>KPIVGRVGSNVIITSGDGVVHAESGGKYG<br>KRNMIYQQLFELKKQDDYYAIIGGWMIG<br>DAFSGTGIREDKSVITGVDSPFAAVRIKTD<br>KLPHPVTLKDIDKMAEDE | 652 | 2VOB | Trypanothione pathway |
| Tryparedoxin peroxidase | *Leishmania major* | MSCGNAKINSPAPSFEEVALMPNGSFKKIS<br>LSSYKGKWVVLFFYPLDFTFVCPTEVIAFS<br>DSVSRFNELNCEVLACSIDSEYAHLQWTL<br>QDRKKGGLGTMAIPMLADKTKSIARSYGV | 199 | **4K1F** | Trypanothione pathway |

| | | | | | |
|---|---|---|---|---|---|
| | | LEESQGVAYRGLFIIDPHGMLRQITVNDM PVGRSVEEVLRLLEAFQFVEKHGEVCPAN WKKGAPTMKPEPNASVEGYFSKQ | | | |
| Squalene synthase | *Leishmania major* | MGFFSDSVAMMRVKWQMRSVKIQVPPEE TDLRFCYDIMNDVSRSFAVVVAQLADQQ LRDAICIFYLVLRALDTLEDDMSVPVDVKL KELPKFHTHTSDMSWCMSGVGEGREREL LAKYPCVSREFKKLKKEYQDVIANICERM ANGMCEFLKRPVVTKDDYNQYCHYVAG LVGHGLTQLFARCGFEDPSLDDDLTSSNH MGLFLQKTNIIRDYYEDIREEPPRMFWPKE IWGTYVTELKELKSESNNAAAVQCLNAM VADALVHVPYIVDYLSALRDPSVFRFCAIP QVMAIATLKEVYNNPDTFQVKVKVSRPES CRIMLKATTLYSSLSMFRDYCVELQEKLD MQDASSVSIANSLAAAIERIDLQLKKCQD VSYTRSLLARYPGLGGQFLLTVMDTVAGF FGGRKEIAGHA | 414 | Swiss Model (**Template used:** 3wca.pdb **Query Coverage:** 82% **Sequence Identity:** 61.83%) | Sterol biosynthetic pathway |
| Squalene monooxygenase | *Leishmania major* | MLYFFAAVLCAVSTLLLLNRTLSRLRLSPA RTSYDYDVIIVGGSIAGPVLAKALSDQGRK VLMVERTLFTKPDRIVGELLQPGGLNALK EVGMKECAETIGMPCHGYVVVDHKGKQ VDLPYRKGASGVSFHFGDFVQSLRSHVFH NCKANVTMVEGTVNNILTEGLSFSERAYG VEYTIAEKYEVPTKPFREDPPKTNPVAATV RKVATAPLVVMCDGGMSKWKSRYQHYT PAYEYHSHFIGLVLKTVRLPKEQRGTVFFG KTGPILSYRLDDNELRLLVDYNKPTLPSLE KQSEWLIQDVAPCLPENMREQFVRVSKDT KSLRSMPMARYPPAFPSIKGYVGIGDHAN QRHPLTGGGMTCCFRDAIRLANSLNGIQS LRSVNQEEMAAIEDKIQAAILNYARYRYT HSCCINLLSWALYSVFSSPALRDACFDYFL CGGNCVTGPMDLLAGLDPNVGSLFFHYC CVMLHGVANVMMRTGAYSESGKQLSNL EKLTNVASFFVDWERMKHAAYLLGKSTQI ALPLAKSEFYSMWRFVDPTSPLANISKRIK TMVYTKQFNGKQRKPVGL | 569 | I-Tasser (Various templates were used including: 6c6n.pdb, 6fho.pdb, 6c6r.pdb) | Sterol biosynthetic pathway |
| Farnesyl pyrophosphate synthase | *Leishmania major* | MAHMERFQKVYEEVQEFLLGDAEKRFEM DVHRKGYLKSMMDTTCLGGKYNRGLCV VDVAEAMAKDTQMDAAAMERVLHDAC VCGWMIEMLQAHFLVEDDIMDHSKTRRG KPCWYLHPGVTAQVAINDGLILLAWATQ MALHYFADRPFLAEVLRVFHDVDLTTTIG QLYDVTSMVDSAKLDAKVAHANTTDYVE YTPFNHRRIVVYKTAYYTYWLPLVMGLLV SGTLEKVDKKATHKVAMVMGEYFQVQD DVMDCFTPPEKLGKIGTDIEDAKCSWLAV TFLTTAPAEKVAEFKANYGSTDPAAVAVI KQLYTEQNLLARFEEYEKAVVAEVEQLIA ALEAQNAAFAASVKVLWSKTYKRQK | 362 | 4JZX | Sterol biosynthetic pathway |

| | | | | | |
|---|---|---|---|---|---|
| Glyceraldehyde-3-phosphate dehydrogenase | *Leishmania major* | MAPIKVGINGFGRIGRMVLQAICDQGLIG NEIDVVAVVDMSTNAEYFAYQMKYDTV HGRPKYTVEAVKSTPSVKTPDVLVVNGHR IKCVKAQRNPADLPWGKLGVDYVIESTGL FTDKLQAEGHIKGGAKKVVISAPASGGAK TIVMGVNQHEYSPTSHHVVSNASCTTNCL APIVHVLTKENFGIETGLMTTIHSYTATQK TVDGVSLKDWRGGRAAAINIIPSTTGAAK AVGMVIPSTKGKLTGMSFRVPTPDVSVVD LTFRSTRETSIQEIDKAIKKAAQTYMKDILG FTNDELVSSDFINDNRSSVYDSKATLQNN LPGEKRFFKIVSWYDNEWGYSHRVVDLVR YMAATDAASAKM | 361 | Swiss Model (**Template used:** 1gyq.pdb **Query Coverage:** 99% **Sequence Identity:** 94.69%) | Glycolytic pathway |
| Triosephosphate isomerase | *Leishmania major* | MSAKPQPIAAANWKCNGTTASIEKLVQV LNEHNISHDVQCVVAPTFVHIPLVQAKLR NPKYVVSAENAIAKSGAFTGEVSMPILKD LGINWVILGHSERRTYYGETDETVAQKVA DACKQGFMVIACIGETLQQREANQTAKV VLSQTSAIATKLPKEAWDQIVLAYEPVWA IGTGKVATPEQAQEVHALLRKWVSEKIGT DVAAKLRILYGGSVSAGNAKTLYMKPDIN GFLVGGASLKPEFRDIIDATR | 251 | Swiss Model (**Template used:** 5cg7.pdb **Query Coverage:** 100% **Sequence Identity:** 91.63%) | Glycolytic pathway |
| Phosphoglycerate kinase | *Leishmania major* | MSLVLKKSIDDATVRDKKVLIRVDFNVPV KNGKITNDFRIRSALPTIQKVLKEGGSCIL MSHLGRPKGARMSDPSPEKGVRGYEEAA TLRPVAARIAELLGQKVEFAPDCLDAAAY ASKLKNGDVLLLENVRFYAEEGSKKEEER DAMAKVLASYADLYVSDAFGTAHRDSAT MTGIPKVLGAGYAGYLMEKEINYFSRVLN NPPRPLVAIVGGAKVSDKIELLDNMLSRIN YLVIGGAMAYTFQKAQGRKIGISMCEEDK LDLAKSLLKKAQERGVQVFLPVDHVCNK EFKAADSPLVTESVDVPDGYMALDIGPRTI HMYEEVIGRCKSAIWNGPMGVFEMPCYS KGTFAVAKAMGTGTQKNGLLSIIGGGDS ASAAELSGEAKNMSHVSTGGGASLELLEG KTLPGVAILTDKDVKERGASCRFAFGVGS PSREACPLRCGHIFGGASIVREIVKLVVALL IGIFIGRRMSTKLIR | 479 | Swiss Model (**Template used:** 16pk.pdb **Query Coverage:** 86% **Sequence Identity:** 73.39%) | Glycolytic pathway |
| Pyruvate kinase | *Leishmania major* | MSQLAHNLTLSIFEPVANHRATRIVCTIGP STQSVEALKGLIQSGMSVARMNFSHGSHE YHQTTINNVRQAAAELGVNIAIALDTKGP EIRTGQFVGGEAVMERGATCYVTTDPAFA DKGTKDKFYIDYQNLSKVVRPGSYIYIDDG ILILHVQSHEDEQTLKCTVTNAHTISDRRG VNLPGCDVDLPAVSAKDCADLQFGVEQG VDMIFASFIRSAEQVGDVRKALGAKGHDI MIICKIENHQGVQNIDSIIEESDGIMVARG DLGVEIPAEKVVVAQKILISKCNVAGKPVI CATQMLESMTYNPRPTRAEVSDVANAVF NGADCVMLSGETAKGKYPNEVVQYMARI | 499 | Swiss Model (**Template used:** 3hqn.pdb **Query Coverage:** 100% **Sequence Identity:** 95.59%) | Glycolytic pathway |

| | | | | | |
|---|---|---|---|---|---|
| | | CLEAQSAVNEYVFFNSIKKLQPIPMSAEEA VCSSAVNSVYETKAKVMVVLSNTGRSARL VAKYRPNCPIVCVTTRLQTCRQLNITQGV ESVFFDAEKLGHDEGKEQRVAMGVGFAK SKGYVQTGDYSVVIHADHKVKGYANQTR ILLVE | | | |
| Phosphoglycerate mutase (2,3-diphosphoglycerate-independent) | *Leishmania major* | MSNLLLRPHKDLPRRKLLIVVMDGLGIGP EDEYDAVHMASTPFMDAQRQNSRHFRSV RAHGTAVGLPTDADMGNSEVGHNALGA GRVALQGASLVDDALKSGEIYTGEGYRYL LGAFTKEGSTLHLIGLLSDGGVHSRDNQI HSIIEHAAKNGAKGIRVHVLYDGRDVPDG SSFRFTEELEAVLARVRQDGCDAAIASGG GRMFVTMDRYDADWSIVERGWKAQVLG DARHFHSAKEAITTFREEDPKVTDQYYPPF VVVDEQGKPLGTIEDGDAVLCVNFRGDR VIEMTRAFEDEDFDKFDRVRVPKVRYAG MMRYDGDLGIPNNFLVPPPKLTRVSEEYL CGTGLQIFACSETQKFGHVTYFWNGNRSG KVDEEHETFKEVPSDRVHFNEQPKMKSAE ITEAAIEALKSGMYDVVRINFPNGDMVGH TGDLRATIAGVEAVDESLAKLKDAVDSIN GVFIVTADHGNSDDMAQRDKKGKPMKD EKGNVLPLTSHTLSPVPVFIGGAGLDPRVA MRTDLPTAGLANVTATFINLLGFEAPEDY EPSLIFVEN | 553 | Swiss Model (**Template used:** 3igy.pdb **Query Coverage:** 100% **Sequence Identity:** 92.21%) | Glycolytic pathway |
| Fructose-bisphosphate aldolase | *Leishmania major* | MSRVTIFQSQLPACNRIKTPYESELIATVKK LTTPGKGLLAADESIGSCTKRFEPIGLSNTE EHRRQYRALMLEAEGLEQYISGVILHDET VSQKASNGKTFPEYLTARGVVPGIKTDMG LCPLLEGAEGEQMTEGLDGYVKRASVYYK KGCRFCKWRNVYKIQNGTVSEPAVRFNA ETLARYAILSQMSGLVPIVEPEVMIDGKHD IDTCQRVSEHVWREVVAALQRHGVIWEG CLLKPNMVVPGAESGQTAAPEQVAHYTV MTLARTMPAMLPGVMFLSGGLSEVQASE YLNAINNSPLPRPYFLSFSYARALQSSALK AWGGKDSGVAAGRRAFLHRARMNSMA QLGKYKRADDDASSSLYVKGNIY | 371 | Swiss Model (**Template used:** 2qap.pdb **Query Coverage:** 100% **Sequence Identity:** 95.96%) | Glycolytic pathway |
| Adenine phosphoribosyltransferase | *Leishmania major* | MSFKEISPNSFLLEDSHPLSQLLKKNYCWY SPVFSPRNVPRFADVSSITESPETLKAIRDFL VQRYRTMSPAPTHILGFDARGFLFGPMIA VELGIPFVLMRKADKNAGLLIRSEPYEKEY KEAAPEVMTIRYGSISKGSRVVLIDDVLAT GGTALSGLQLVEASDAMVVEMVSILCIPFL KAAEKIHSTGHSRYKDIKFISLLSEEALTED NCGDSKNYTGPRVVSCGDVLSKHSQ | 237 | Swiss Model (**Template used:** 1qb7.pdb **Query Coverage:** 98% **Sequence Identity:** 92.27%) | Purine salvage pathway |
| Xanthine phosphoribosyltransferase | *Leishmania major* | MLPNHSCKGFVDAQGRVFVDGREYPMAS GIVATEDVIQANIKAMANTIAKDYKSLSH RDVRLSPGTAATAEAAEAPISYDNPLIIISV | 238 | Swiss Model | Purine salvage pathway |

| | | | | | |
|---|---|---|---|---|---|
| | | LKGSYIFTSDFIRYLGDCGLPHVVDFVRLA<br>SYNSDTTSTGQISMLAGLRFENLRGKHVLI<br>VEDVCDSGRTLRFLRDYIMEKFQPKSVKTL<br>VMVNKEQAARKLDFDPEYFCLAGPNKYI<br>VGYGFEVNDRYRDLRHIFILRDGEATRYP<br>AKL | | (**Template used:** 6mxc.pdb **Query Coverage:** 92% **Sequence Identity:** 56.36%) | |
| Deoxyhypusine Hydroxylase | *Leishmania major* | MSASNSCTVEEVRKEYAKLLDPQEPLDSR<br>MRELYRLKEDCLKTVAGVTVILEAIDTTDS<br>VLLQHELAYNAGQSGREEAVPELERILRTT<br>SYDVVTRHEAAEALGAIGSPLALQVLETH<br>SAPTTEPEASIRETCELALARIAMKETKGD<br>AAVAPPSGCEFVSVDPSPAFSALYSSTDEP<br>VPLTVEELEAVLLDTSGRTRLFRRYMAMF<br>TLRNLATEAAVAALCRGLREDTISALFRH<br>EVAFVLGQLERPSSQPALIAALKDEEEAP<br>MVRHEAAEALGAIADPATLPVLESYATH<br>HEPIVRDSCVVALEMHKYWAHFNSLAHQ<br>QQEA | 325 | I-Tasser (Various templates were used including: 4d4z.pdb, 6fsq.pdb) | Hypusine biosynthetic pathway |
